# Structure of the inhibitor complex of old yellow enzyme from *Trypanosoma cruzi*

**DOI:** 10.1107/S0909049510033595

**Published:** 2010-11-12

**Authors:** Keishi Yamaguchi, Naoki Okamoto, Keiji Tokuoka, Shigeru Sugiyama, Nahoko Uchiyama, Hiroyoshi Matsumura, Koji Inaka, Yoshihiro Urade, Tsuyoshi Inoue

**Affiliations:** a Department of Applied Chemistry, Graduate School of Engineering, Osaka University, Yamada-Oka 2-1, Suita, Osaka 565-0871, Japan; b National Institute of Health Sciences (NIHS), Tokyo 158-8501, Japan; c MARUWA Foods and Biosciences, Tsutsui-cho 170-1, Yamatokoriyama, Nara 639-1123, Japan; d Department of Molecular Behavior Biology, Osaka Bioscience Institute, Osaka 565-0874, Japan

**Keywords:** X-ray structure, inhibitor complex, prostaglandin synthase

## Abstract

Old yellow enzyme (OYE) is an NADPH oxidoreductase which contains flavin mononucleotide as prosthetic group. The X-ray structures of OYE from *Trypanosoma cruzi* (TcOYE) which produces prostaglandin (PG) F_2α_ from PGH_2_ have been determined in the presence or absence of menadione. The binding motif of menadione, known as one of the inhibitors for TcOYE, should accelerate the structure-based development of novel anti-chagasic drugs that inhibit PGF_2α_ production specifically.

## Abstract 2

The structures of old yellow enzyme from *Trypanosoma cruzi* which produces prostaglandin F_2α_ from PGH_2_ have been determined in the presence or absence of menadione.

## 1. Introduction

Prostaglandin (PG) F_2α_ synthase (PGFS) is the enzyme which produces PGF_2α_ from the PGH_2_, and was first isolated from mammals (Watanabe *et al.*, 1985 ▶). Mammalian PGFSs belong to aldo-ketoreductases (Bohren *et al.*, 1989 ▶; Bruce *et al.*, 1994 ▶), which are NAD(P)(H)-dependant oxidoreductase. In mammals, PGF_2α_ is a potent mediator of various physiological processes (Narumiya *et al.*, 1999 ▶; Glew, 1992 ▶; Samuelsson, 1979 ▶) including vasculartone, constriction of uterine muscle (Bygdeman *et al.*, 1970 ▶) and pulmonary arteries (Oliw *et al.*, 1983 ▶; Mathe *et al.*, 1977 ▶), and induction of luteolysis during the estrous cycle and prior to parturition (Horton & Poyser, 1976 ▶; McCracken *et al.*, 1972 ▶). During pathological processes in mammals, PGF_2α_ overproduction causes ovarian dysfunction and miscarriage (Dubois *et al.*, 1998 ▶; Mutayoba *et al.*, 1989 ▶; Davies *et al.*, 1984 ▶).

PGF_2α_ is produced not only in mammals but also in parasitic protozoa which causes serious infections, such as *Plasmodium falciparum* (Kubata *et al.*, 1998 ▶), *Trypanosoma brucei* (Kubata *et al.*, 2000 ▶; Okano *et al.*, 2002 ▶; Kilunga *et al.*, 2005 ▶) and *Leishmania major* (Kabututu *et al.*, 2002 ▶, 2003 ▶). Owing to their identification, pathological relationships of parasitic PGFSs with human disease have begun to emerge. For example, it is thought that PGFS from *Trypanosoma brucei* (TbPGFS) plays a role in pathogenesis trypanosomiasis because African trypanosomiasis is characterized by miscarriage owing to PGF_2α_ overproduction correlated with parasitemia peaks (Mutayoba *et al.*, 1989 ▶). Therefore, the study of parasitic PGFS is quite significant because it can lead to investigation of the pathological roles and development of the inhibitor of PGFS for remedies.

Chagas disease, caused by *Trypanosoma cruzi*, affects more than 20 million people and poses a major public health and economic problem in South America (World Health Organization, 1990 ▶). This situation has been worsened by the lack of effective vaccines and undesirable side effects of anti-chagasic drugs, such as nifurtimox and benznidazole (Aldunate & Morello, 1993 ▶; Henderson *et al.*, 1988 ▶; Docampo & Moreno, 1986 ▶; Docampo, 1990 ▶), in addition to the emergence of parasite resistance to these drugs. Therefore, the development of a new chemotherapeutic treatment is an urgent need.

PGFS from *T. cruzi* was identified by Kubata *et al.* and it was found that this enzyme belonged to old yellow enzyme (OYE) (Kubata *et al.*, 2002 ▶). OYE was originally isolated from brewers’ bottom yeast in the 1930s, and the first protein requiring a vitamin B_2_-derived molecule, flavin mononucleotide (FMN), of its catalysis (Warburg, 1933 ▶; Schopfer & Massey, 1991 ▶). The previously detected OYE families from bacteria (Warburg, 1933 ▶; Matthews & Massey, 1969 ▶), yeast (Schaller & Weiler, 1997 ▶) and plants (French *et al.*, 1996 ▶; Blehert *et al.*, 1999 ▶) are able to reduce a variety of compounds, such as unsaturated aldehydes and ketones (French & Bruce, 1994 ▶; Vaz *et al.*, 1995 ▶), nitro-esters (French *et al.*, 1996 ▶; Snape *et al.*, 1997 ▶; Blehert *et al.*, 1999 ▶; Meah & Massey, 2000 ▶) and nitro-aromatic substrates (French *et al.*, 1998 ▶; Pak *et al.*, 2000 ▶; Williams *et al.*, 2004 ▶).

**Figure 1:**
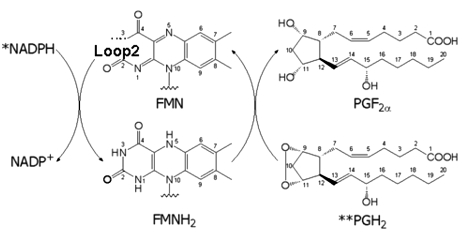
Reduction of PGH_2_ to PGF_2α_ by TcOYE. FMN (cofactor of TcOYE) is reduced to FMNH_2_ by intravital nicotinamide adenine dinucleotide phosphate (NADPH), and the product PGF_2α_ is subsequently generated by FMNH_2_.

OYE from *T. cruzi* (TcOYE) catalyzes prostaglandin F_2α_ synthesis (Fig. 1 ▶) as well as the reduction of many kinds of trypanocidal drugs, such as naphtoquinone and nitrohetero­cyclic compounds (Kubata *et al.*, 2002 ▶). By electron spin resonance it was found that these compounds can undergo either one- or two-electron reduction. Naphtoquinone, such as menadione (*K*_m_ = 0.82 µ*M*) or β-lapachone (*K*_m_ = 0.17 µ*M*), is reduced by one-electron reduction, generating semiquinone radicals which cause the cell death in susceptible parasites, while nitroheterocyclic compounds, such as 4-nitroquinoline-*N*-oxide (*K*_m_ = 9.5 µ*M*) or nifurtimox (*K*_m_ = 19.0 µ*M*), are reduced by two-electron reduction (Kubata *et al.*, 2002 ▶). Therefore, three-dimensional information of the structure in complex with the quinine compounds is valuable for the design of novel anti-chagasic drugs by which semiquinone radicals are more efficiently generated in *T. cruzi*.

Here we report the first X-ray structures of TcOYE which produces PGF_2α_ from PGH_2_ in the presence or absence of menadione. The structural analysis of TcOYE/FMN/menadione ternary complex has provided the binding motif of menadione at the active site. These structures are useful for further drug design for Chagas disease.

## 2. Materials and methods

### 2.1. Protein expression and crystallization

Chemicals were obtained from Sigma-Ardrich at the highest available purity. TcOYE was overexpressed in *E. coli* and purified, and TcOYE/FMN binary complex was crystallized according to previously reported methods (Kubata *et al.*, 2002 ▶; Sugiyama *et al.*, 2007 ▶). These crystals were flash-cooled in liquid nitrogen with cryo-protectant solution containing 3% (*v*/*v*) 2-methyl-2,4-pentanediol. The X-ray diffraction data were obtained up to 1.70 Å resolution at SPring-8 beamline BL41XU. The diffraction data were processed and scaled using the program *HKL2000* (Otwinowski & Minor, 1997 ▶).

### 2.2. Structure determination and refinement

The structure of TcOYE was determined by molecular replacement with the program *AMoRe* (Navaza, 2001 ▶) using the structure of morphinone reductase from *Pseudomonas putida* (Protein Data Bank code 1gwj) (Barna *et al.*, 2002 ▶) as a search model. Crystallographic refinement was carried out with the program *CNS* (Brünger *et al.*, 1998 ▶). The refinement procedure included simulated annealing, positional refinement, restrained temperature factor refinement, and maximum-likelihood algorithms as provided by the *CNS* program. Electron density maps based on the coefficients of 2*F*_o_ − *F*_c_ and *F*_o_ − *F*_c_ were used to build the atomic models in *Coot* (Emsley & Cowtan, 2004 ▶). Water molecules were inserted manually and then checked by inspecting the *F*_o_ − *F*_c_ map. FMN (cofactor) and menadione (ligand) were refined using atomic parameters extracted from the HIC-UP server (Kleywegt & Jones, 1998 ▶), and could be inserted into the clearly defined electron density model after some cycles of refinement.

### 2.3. Preparation of TcOYE complexed with menadione

In order to prepare the complex crystals with menadione, the crystals of the TcOYE/FMN complex were soaked in a menadione solution for 16 h and then the crystals were flash frozen in liquid nitrogen. The X-ray diffraction data were obtained up to 2.5 Å resolution by using Ultrax18 (Rigaku). Data collection, structure determination and refinement were carried out by the same methods as described above.

## 3. Results and discussion

### 3.1. Overall structure of TcOYE

The structure of TcOYE/FMN binary complex was determined by molecular replacement methods using that of morphinone reductase (Barna *et al.*, 2002 ▶) as the searching model and refined at 1.7 Å resolution with final *R*_cryst_/*R*_free_ values of 18.5%/23.2%. Our crystallographic properties and refinement statistics of the structure are presented in Table 1 ▶.

**Figure 2:**
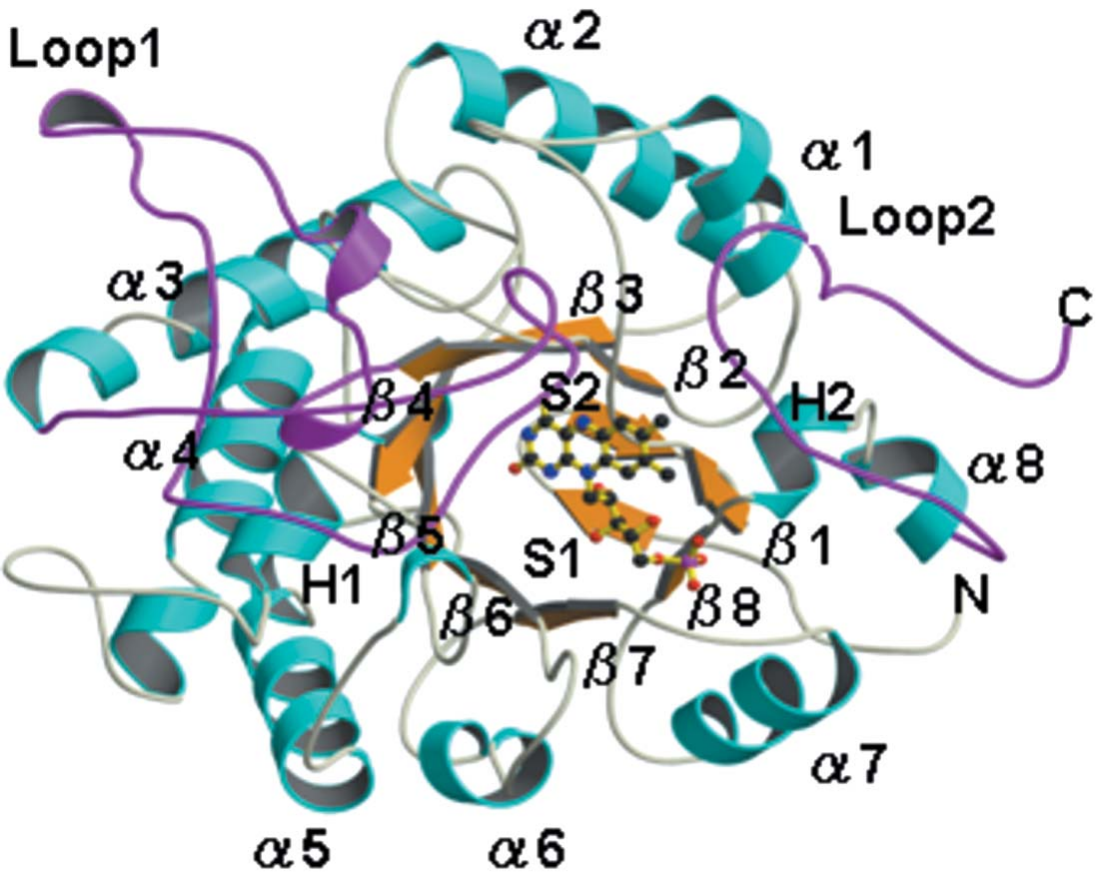
The overall structure of TcOYE. The α-helices (cyan) and β-sheets (orange) are separated among them by loops (light yellow). Loop 1 and Loop 2 are shown in magenta. FMN is shown as a ball-and-stick model. The image was created by using *MOLSCRIPT* (Kraulis, 1991 ▶) and *RASTER3D* (Merritt & Murphy, 1994 ▶).

TcOYE, like other members of the OYE family, folds into an (α/β)_8_ barrel in which the cylindrical core composed of eight parallel β-strands (β1–β8) is surrounded by eight α-helices (α1–α8). In addition, an N-terminal β-hairpin (S1 and S2) closes the bottom of the barrel and two extra helices (H1 and H2) lie in the loops connecting β4 to α4 and β8 to α8, respectively (Fig. 2 ▶). The loop regions from 105 to 165 (Loop 1) and 351 to 379 (Loop 2) exhibit the relatively higher *B*-factors of 21.3 and 18.8 Å^2^, respectively, while the overall *B*-factor of TcOYE without those two loop ranges was calculated to be 13.1 Å^2^. This difference in the *B*-factor shows the flexibility of these two loops.

### 3.2. FMN binding site

**Figure 3:**
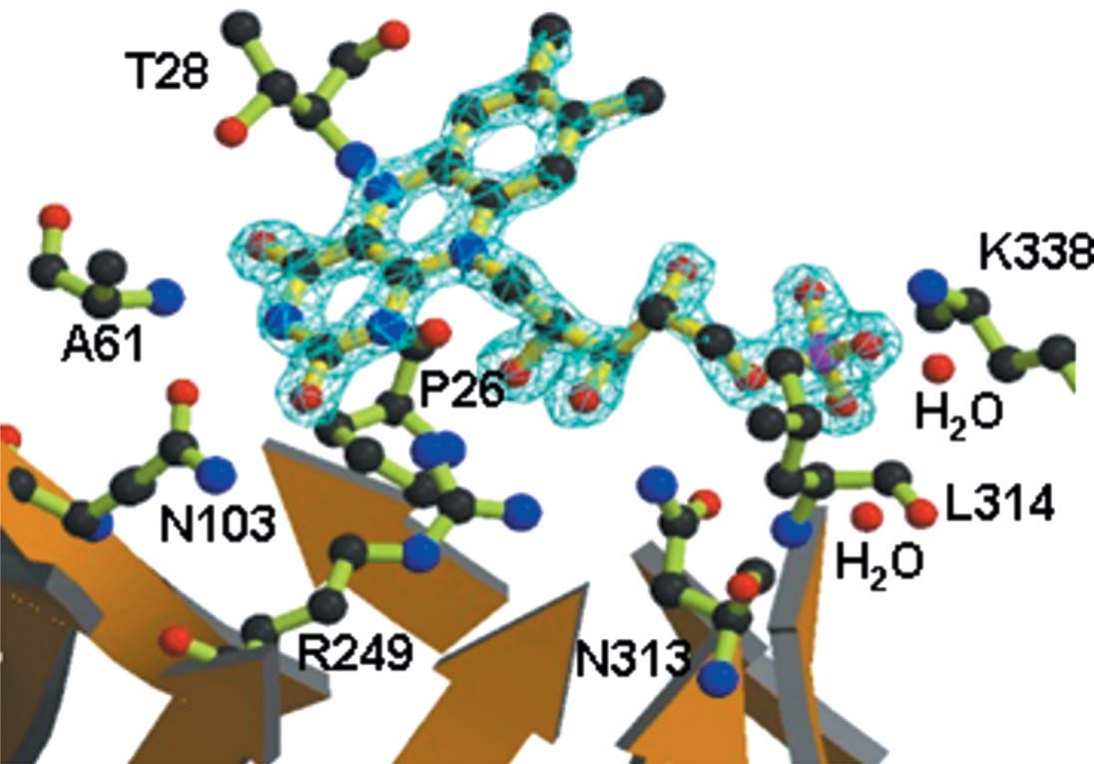
FMN binding model with 2*F*_o_ − *F*_c_ omit map. The FMN electron density is calculated at 1.7 Å and contoured at 2.5 σ. O, N and P atoms are shown in red, blue and magenta, respectively. Labelled residues indicate those involved in FMN binding. The figure was drawn using the programs *MOLSCRIPT* and *RASTER3D*.

FMN is tightly bound at the C-terminal ends of the eight β-strands in the barrel and exhibits quite low *B*-factors (Fig. 3 ▶). Most amino acid residues which bind to the riboflavin moiety of FMN are highly conserved among the different known OYE structures, except Ala61. Ala61 in TcOYE was conserved as alanine or glycine among the known OYE structures; however, the amide nitrogen atom of the corresponding amino acid interacts with O4 of FMN. On the other hand, three residues bound to the phosphate O atoms in FMN are not conserved: the side chain of Asn313 and the main chains of Leu314 and Lys338 interact with the phosphate O atoms through water molecules.

### 3.3. The binding motif of menadione

**Figure 4:**
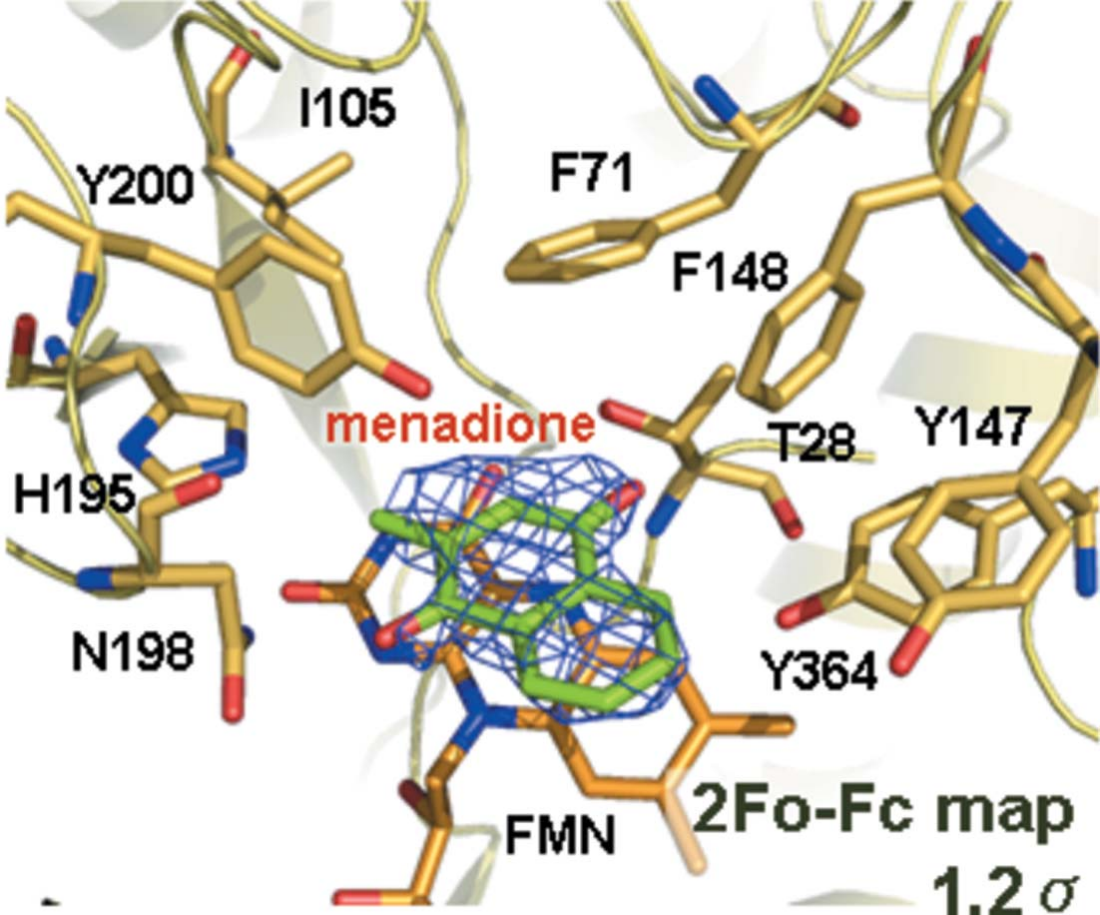
The binding site of menadione. Menadione (green) is positioned above the isoalloxazine ring of FMN. The menadione electron density is calculated at 2.5 Å and contoured at 1.2 σ. The figure was drawn using the program *PyMOL* (DeLano, 2005 ▶).

In order to develop more effective inhibitors we have solved the structure of TcOYE/FMN/menadione ternary complex at 2.5 Å resolution (Table 1 ▶). The whole structure of TcOYE does not change even upon binding of menadione. The binding motif of menadione is defined in the difference electron density maps even at low occupancy (Fig. 4 ▶). The cause of its low affinity (*K*_m_ = 0.82 µ*M*) is assumed to be because the ligand is stabilized by only a π–π interaction with the isoalloxazien ring of FMN. To increase the affinity of menadione, interactions with His195, Asn198 or Tyr 364 should be designed in the future.

## 4. Conclusion

We have solved the structures of TcOYE in the presence or absence of menadione. The binding motif of the inhibitor at the active site of TcOYE has been elucidated from TcOYE/FMN/menadione ternary complex. Future work is in progress to obtain the structure in complex with other inhibitors to elucidate the reaction mechanism of TcOYE in detail, which can be expected to lead to the development of heroic anti-chagasic drugs.

## Figures and Tables

**Table 1:** Data collection and refinement statistics for TcOYE/FMN and TcOYE/FMN/menadione Values in parentheses are for the highest resolution shell.

	TcOYE/FMN	TcOYE/FMN/menadione
Beamline	SPring-8 BL41XU	Ultrax18
Space group	*P*2_1_	*P*2_1_
Cell constants (Å, °)	*a* = 56.29, *b* = 78.79, *c* = 78.80, β = 93.37	*a* = 47.58, *b* = 53.66, *c* = 120.00, β = 90.004
Resolution range (Å)	50.0–1.70 (1.76–1.70)	50.0–2.5 (2.54–2.50)
No. of molecules per asymmetric unit	2	2
*V*_M_ (Å^3^ Da^−1^)	2.1	1.8
*V*_solv_ (%)	41	35
No. of measured reflections	424814	70984
No. of unique reflections	75469	20908
*I*/σ(*I*)	6.3	6.8
*R*_merge_ (%)†	7.0 (27.4)	3.5 (6.2)
Completeness (%)	100.0 (100.0)	98.1 (95.0)
*R*_cryst_ (%)‡	18.5	33.2
*R*_free_ (%)§	23.2	40.9
R.m.s. deviations		
Bonds (Å)	0.005	0.01
Angles (°)	1.4	1.7

† *R*_merge_ = Σ|*I*(*k*) − *I*|/Σ*I*(*k*), where *I*(*k*) is the value of the *k*th measurement of the intensity of a reflection, *I* is the mean value of the intensity of that reflection, and the summation is over all measurements.

‡ *R*_cryst_ = Σ||*F*_o_| − |*F*_c_||/Σ|*F*_o_|, calculated from 90% of the data, which were used during the course of the refinement.

§ *R*_free_ = Σ||*F*_o_| − |*F*_c_||/Σ|*F*_o_|, calculated from 10% of the data, which were obtained during the course of the refinement.
